# The Usefulness of Procalcitonin in the Diagnosis of Appendicitis in Children: A Pilot Study

**DOI:** 10.1155/2012/317504

**Published:** 2012-12-05

**Authors:** Abu N. G. A. Khan, Abdel Sawan, Antonios Likourezos, Mark Schnellinger, Estavan Garcia

**Affiliations:** ^1^Department of Emergency Medicine, Maimonides Medical Center, 4802 Tenth Avenue, Brooklyn, NY 11219, USA; ^2^Division of Pediatric Emergency Medicine, Morgan Stanley Children Hospital of New York-Presbyterian, 622 West 168th Street, PH 137-1, New York, NY 10032, USA

## Abstract

*Objective*. To assess the predictive value of procalcitonin in detecting acute appendicitis (AP) in children, and to determine a cutoff value of procalcitonin which can safely include/exclude the diagnosis of acute appendicitis in children with acute abdominal pain. *Methods*. Prospective cohort study of children aged 5–17 years presenting to the emergency room with right lower quadrant (RLQ) tenderness and strong suspicion for acute AP. In addition to standard diagnostic workup for acute AP, a quantitative procalcitonin level was measured using immunoluminometric assay. Recursive partitioning model was used to assess the usefulness of procalcitonin in the diagnosis of appendicitis. *Results*. Of the 50 children studied, 48% were diagnosed to have AP. The mean procalcitonin level was higher among the children with appendicitis (*P* = 0.3). Using the recursive partitioning model, we identified a cutoff value of procalcitonin level of 0.39 with a likelihood ratio presence of appendicitis 3.25 and absence of appendicitis 0.8. None of the study subjects with procalcitonin level <0.39 and WBC count of <6.76 K had appendicitis. *Conclusions*. In conjunction with the clinical symptoms, a procalcitonin level and WBC count could be a strong predictor of acute appendicitis in children.

## 1. Introduction

Abdominal pain is one of the most frequently encountered complaints in the pediatric emergency room (ER). The approach to a child with abdominal pain often presents a dilemma to the pediatric ER staff. The pediatric ER physician and surgeon must be able to develop a differential diagnosis based on the clinical presentation of the child, in order to formulate a final diagnosis. Amongst the numerous etiologies of abdominal pain in children, acute appendicitis is one of the most common one that requires immediate intervention.

Appendicitis affects 6% of the population [1, 2]. Morbidity and mortality rates are related to the time from the first onset of symptoms to the definitive diagnosis. Complications of misdiagnosing appendicitis include intraabdominal abscess, wound infection, adhesion formation, bowel obstruction, and infertility. 

The ability to diagnose acute appendicitis, reliably, is not clear-cut. Along the way, various predictive indicators have been researched to help make the diagnosis of acute appendicitis easier. In general, any patient with abdominal pain and more specifically, right lower quadrant tenderness, is approached with the goal of eliciting enough evidence to be able to rule in or out acute appendicitis. Information is gathered via clinical examination, laboratory studies, radiological imaging, surgical consults and sometimes even exploratory surgery to try to determine if the child has acute appendicitis. Within the laboratory studies, classically, a WBC count and ESR/CRP levels are the most widely used parameters [3]. 

There have been attempts to improve these results using ultrasound or CT scans to reduce the amount of missed appendicitis cases and unnecessary operations. Ultrasound has a high sensitivity, but it is technician dependent and its value is limited in the evaluation of a ruptured appendix or abnormally located appendix, such as a retrocecal appendix. Computed tomography is more sensitive than ultrasound with higher comparable specificity [4, 5]. However, recently the FDA has raised concerns about the increased use of CT scans exposing the public to risky levels of radiation. Children, in particular, are at a greater risk than adults because they have more rapidly dividing cells and a longer life expectancy, giving them greater odds of developing cancer [6]. According to the National Cancer Institute, the approximate dose of radiation from a pediatric abdominal CT scan is 6–15 mSv. Since radiation of 50–200 mSv has been found to increase cancer risks, the National Cancer Institute stresses that it is important to minimize CT radiation doses especially when it comes to children [7, 8]. The National Research Council Committee on the Biological Effects of Ionizing Radiation estimates that children less than 10 years of age are several times more sensitive to radiation than middle-aged adults.

Procalcitonin (116 amino acid propeptide of calcitonin) is normally produced in the C-cells of the thyroid gland. Normal levels in healthy individuals are very low (<0.1 ng/mL) [9]. Procalcitonin has been introduced as an early marker of severe systemic bacterial infection and inflammation [10–13]. Its level is related to the severity of infection. In patients with sepsis, the levels increase to more than several hundred nanograms per milliliter. Its half-life is approximately 24 hours in serum [14]. Although estimation of quantitative procalcitonin level is cumbersome, a quick qualitative test could be easily developed for point of care testing. 

In search of a diagnostic marker to improve the accuracy of clinical diagnosis of appendicitis and to reduce dependency on abdominal CT scan, we decided to look into the usefulness of procalcitonin levels in diagnosing appendicitis in children with right lower quadrant pain/tenderness in the pediatric emergency room setting. 

The objectives of this study were to (a) determine the procalcitonin level in patients with suspected acute appendicitis by correlating it with the histopathologic diagnosis and (b) identify a cutoff value of procalcitonin which can safely include/exclude the diagnosis of acute appendicitis in children with acute abdominal pain. 

## 2. Methods

 This prospective study was conducted in a Pediatric Emergency Department (PED) of an urban tertiary care medical center. A convenience sample of children ages 5–17 years presenting to the PED for evaluation of right lower quadrant abdominal pain without signs of obvious gastroenteritis, no recent diagnosis or workup for appendicitis, no chronic hematologic, immunologic or gastrointestinal disease, and nonpregnant were enrolled for the study. 

Although the literatures suggest the prevalence of appendicitis in children with right lower quadrant pain varies from 0.2 to 0.8 percent [15, 16]. There were no clear data available for the prevalence of appendicitis in children who meet the inclusion criteria in this study population. In addition, no data were available on the standard procalcitonin level (cutoff value) for the presence or absence of appendicitis. We made the following assumptions to calculate the sample size: prevalence of appendicitis among patient who meets the clinical criteria is 0.3; the test has 80% sensitivity and 90% specificity. Using the Normogram [17], a sample size of 480 was estimated to have a fairly precise confidence interval of 0.05. To reduce the cost, we decided to conduct a pilot study with 20% of the required sample size.

All attending physicians covering the ED were trained to enroll patients for this study. Subjects who met eligibility criteria were approached to be enrolled by the clinician. Participation in the study was voluntary. Informed consent was obtained from the legal guardian. In addition to the normal laboratory workup for abdominal pain, one milliliter (mL) of extra blood in a small red top tube was collected for the study purpose. Quantitative procalcitonin level was estimated using an immunoluminometric assay kit [18]. As per the manufacturer's guidelines, we obtained two back-to-back measurements from the same sample and use the mean of the two measurements as the official result. The specimen was discarded if there was more than 10% variation between the estimates. Data points also include demographics (age, sex, race); clinical presentations such as fever, vomiting, diarrhea; characteristics of abdominal pain including duration, location, and progression; abdominal examination such as tenderness, location, presence or absence of rebound tenderness; as well as lab results, such as WBC count, ANC, CT scan/ultrasound (if ordered). The decision to obtain additional imaging or diagnostic methods was left to the discretion of the clinician taking care of the patient.

The presence of appendicitis was confirmed by histopathologic evidence of appendicitis, such as swelling, signs of inflammation and presence or absence of perforation. Absences of appendicitis were confirmed by telephone followups in 24 hours and 2 weeks after discharge from the hospital.

Descriptive statistics were performed using SPSS Version 15.0 [19]. A recursive partition model was fitted using CART (classification and regression trees) 5.0 by Sanford Systems [20]. We used the histopathologic diagnosis of appendicitis as the outcome and entered pertinent clinical observations (including sex, fever, vomiting, diarrhea, and right lower quadrant abdominal tenderness) and labs results (WBC count, ANC, and procalcitonin level) as variables to obtain a cut of value of the highest negative predictive value.

The study was approved by the medical center's institutional review board (IRB). Consent was obtained from each child's parent. All data were kept confidential and used only for research purposes. The participant's privacy was maintained by giving each a unique ID number in the database for analyses. Accesses to the database files were protected by encryption and a password. All data forms were secured in a locked file cabinet, and access was limited to the investigators and other agencies as permitted by the law.

## 3. Results

Fifty-six patients were approached during the study period. Four patients refused to participate in the study. Two patients were excluded; one due to defective specimen and the other had acute right ovarian torsion. Among the fifty children (age group 11 ± 3.2 years) enrolled in the study, 22 patients (44%) had confirmed appendicitis by histopathology. Demographic and clinical characteristic of the patients are shown in Table 1.

Eighty percent had an abdominal CT scan done in the ED. All patients with appendicitis and 18/28 nonappendicitis patients had abdominal CT with oral & intravenous contrast in the ED. Twenty four (24/50; 48%) children diagnosed to have appendicitis, 5/50 mesenteric adenitis, 3/50 gastroenteritis, 18/50 others. Twenty-two (22/50; 44%) patients were discharged home after evaluation. One (1/22) discharged patient was readmitted after 48 hours and subsequently diagnosed to have appendicitis. All patients diagnosed to have appendicitis went for surgery within 24 hours. Twenty-two (22/24; 92%) had confirmed appendicitis by histopathology. Six patients (6/22; 27%) had perforated appendicitis and 2/6 had clinical signs of peritonitis in the ED. The mean procalcitonin level (ng/mL) was higher among the children with appendicitis (1.12 ± 3.28 versus 0.45 ± 1.12; *P* = 0.3). 

**Figure 1:**
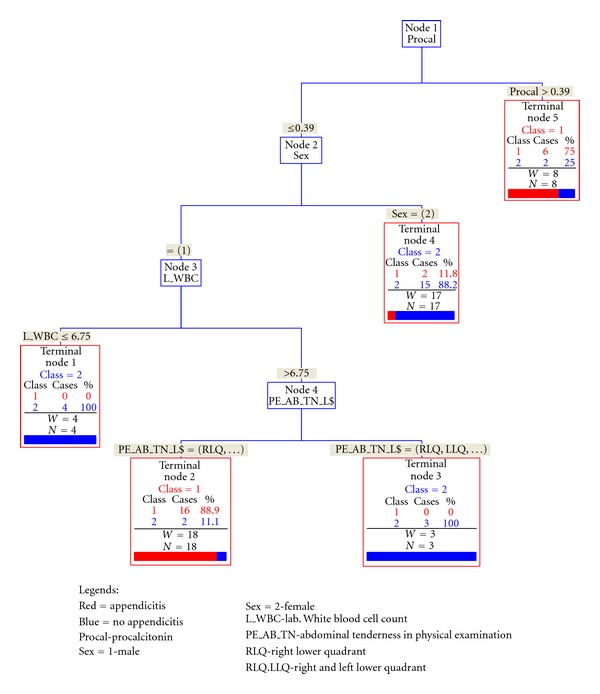
Recursive partitioning model CART.

In the recursive-partitioning model, in addition to procalcitonin level, sex, WBC count, and right lower quadrant tenderness were found to be strongly associated with the final outcome (presence or absence of appendicitis). We identified a cutoff value of procalcitonin level of 0.39 ng/mL with a sensitivity of 0.25 and specificity of 0.92, positive likelihood ratio 3.25 and negative likelihood ratio of 0.8. Notably, male patients with procalcitonin level <0.39 and WBC count of <6.76 K had a specificity of 100% (Figure 1). 

## 4. Discussion

We found that the mean procalcitonin level was higher among the children with appendicitis (1.12 ng/mL ± 3.28 versus 0.45 ng/mL ± 1.12), but it was not statistically significant. This indicates that procalcitonin level may not be a good independent predictor of presence or absence of appendicitis. This finding echo the results of the other three published studies comparing procalcitonin and appendicitis in children, with sample sizes ranging from 43 to 212 children [21–23]. 

However, our data indicates that a procalcitonin of >0.39 is a reasonably good predictor of appendicitis. In conjunction with clinical finding and WBC count [24], procalcitonin level could be very helpful in diagnosing as well as ruling out of appendicitis in children.

Our study had several limitations. First it is a pilot study, vulnerable for type II error. Second, like most of the ED clinical study we used convenience sample rather than a continuous sample over time. Third, since our goal was to catch as many as possible appendicitis patients, we used stringent selection criteria that lead to a relatively sicker population (approximately 48% appendicitis).

## 5. Conclusions

Although we are presenting a pilot data, we found a cutoff value of procalcitonin level with a reasonable likelihood ratio. And in conjunction with the clinical symptoms and WBC count, a procalcitonin level could be very useful in diagnosing or ruling out acute appendicitis in children. Further study with adequate sample size is indicated to determine the validity of our observation.

## Figures and Tables

**Table 1:** Demographics and clinical features.

	Appendicitis	No appendicitis	Significance
	*N* = 22	*N* = 28	(*P* value)
Demographics			
Age: (Mean ± SD)	11.25 ± 3.13	10.78 ± 3.32	0.68
Sex: Male : Female	17 : 12	5 : 16	0.023
Race: Cauc: Hisp: Other	7 : 1 : 1	1 : 2 : 5	0.052
Symptoms			
Abdominal pain—Duration	40 ± 32	25 ± 5	0.27
Abdominal pain locations			
RLQ	82%	60%	0.153
LLQ	4.5%	0%	
Both	0%	18%	
Epigstric	9%	18%	
Diffuse	4.5%	4%	
Fever	41%	36%	0.269
Vomitting	73%	61%	0.279
Decreased appetite	86%	68%	0.19
Physical examination			
Temperature (oral)	99.1 ± 1.84	98.7 ± 1.3	0.91
RLQ tenderness	100%	93%	0.49
RLQ guarding	59%	36%	0.15
RLQ rebound	68%	32%	0.02
Bowel sound	86%	100%	0.08
Laboratory values			
WBC	15.5 ± 5.0	10.57 ± 4.4	0.001
ANC	12789 ± 5872	7636 ± 4527	0.013
Radiologic studies			
Abdominal CT (+AP)	94%	7%	0.000
